# The E3 Ubiquitin Ligase TRIM25 Inhibits Tembusu Virus Replication *in vitro*

**DOI:** 10.3389/fvets.2021.722113

**Published:** 2021-09-14

**Authors:** Han Kaikai, Dongmin Zhao, Yuzhuo Liu, Qingtao Liu, Xinmei Huang, Jing Yang, Lijiao Zhang, Yin Li

**Affiliations:** ^1^Key Laboratory of Veterinary Diagnosis, Key Laboratory of Veterinary Biological Engineering and Technology, Ministry of Agriculture, Institute of Veterinary Medicine, Jiangsu Academy of Agricultural Sciences, Nanjing, China; ^2^Institute of Life Sciences, Jiangsu University, Zhenjiang, China; ^3^College of Veterinary Medicine, Nanjing Agricultural University, Nanjing, China; ^4^Jiangsu Key Laboratory for Food Quality and Safety-State Key Laboratory Cultivation Base of Ministry of Science and Technology, Nanjing, China

**Keywords:** duck, Tembusu virus, TRIM25, tissue distribution, viral replication

## Abstract

Duck Tembusu virus (DTMUV) is a newly emerging pathogenic flavivirus that has caused significant economic losses to the duck industry in China since 2010 due to egg production losses and neurological dysfunction. DTMUV is a public health concern because the infection spreads rapidly among birds. Retinoic acid-inducible gene-I (RIG-I)serves as an innate immune sensor and plays a key role in host antiviral defenses. Tripartite motif-containing protein 25 (TRIM25), an E3 ubiquitin ligase, is pivotal for RIG-I ubiquitination and activation. In addition, TRIM25 acts as an interferon-stimulated gene and mediates the antiviral activity. However, the effect of duck TRIM25 on DTMUV has not been assessed. Herein, we reportthe antiviral function of TRIM25 against DTMUV. First, we constructed the pcDNA3.1-c-myc-duTRIM25 plasmid. TRIM25 has a 2052 bp open reading frame that encodes a predicted 684 amino acid protein consisting of a RING finger domain, a B-box domain, a coiled-coil domain, and a PRY/SPRY domain. The protein sequence identity with chicken, mouse, and human TRIM25 is 69.7, 47.8, and 48.3%, respectively. TRIM25 was upregulated in BHK-21 cells, duck embryo fibroblasts, and 293T cellsupon DTMUV infection. The expression of viral RNA and proteins was significantly lower in cells over expressing TRIM25 than in control cells. Furthermore, siRNA-mediated silencing of TRIM25 increased the production of viral progeny. These results help elucidate the molecular mechanisms underlying the host response to DTMUV infection and suggest potential control measures for DTMUV outbreaks.

## Introduction

In April 2010, an outbreak of duck Tembusu virus (DTMUV), also known as BYD virus, occurred in duck farming regions in eastern China (1, 2). Infected ducks exhibit a decrease in weight gain and egg production and may present several symptoms, including diarrhea, high fever, rhinorrhea, acute anorexia, antisocial behavior, and paralysis (3, 4), all of these caused significant economic losses to the duck industry. DTMUV is an enveloped virus with a single-stranded, positive-sense RNA encoding 10.5 kb-long genome. The genome has an open reading frame encoding a large protein (5, 6) processed by viral and cellular proteases into three structural proteins (capsid, prM/M, and envelope) and seven non-structural proteins (NS1, NS2A, NS2B, NS3, NS4A, NS4B, and NS5) (7). Viral particle assembly requires structural proteins, and viral RNA replication is supported by non-structural proteins and counteracts the antiviral immune response of the host (8).

Although diagnostic methods and vaccines for DTMUV have been successfully developed and are utilized in the clinic, current knowledge regarding the etiology and immunology of DTMUV is limited. Knowledge about virus-host interactions is crucial to understand the pathogenesis of viral infections. Viruses rely on the host for RNA replication and protein synthesis and may cause dramatic changes in host cell morphology, transcription and translation patterns, cytoskeleton, cell cycle, innate immune response, apoptosis pathways, and inflammatory and stress responses (9).

In the previous study, tripartite motif-containing protein 25 (TRIM25) was significantly upregulated at 24 h post-infection (hpi) with DTMUV in DF-1 cells (10). TRIM proteins are RING-type E3 ligases that play critical regulatory roles in innate immune signaling pathways. These proteins present a conserved domain containing an N-terminal TRIM comprising a catalytic RING domain, one or two B-box domains, and a coiled-coil domain that mediates dimerization (11). TRIM25 mediates the ubiquitination and activation of N-terminal caspase recruitment domains (CARDs) of RIG-I (12). This activation initiates an antiviral signaling cascade *via* MAVS/VISA/IPS-1, leading to the phosphorylation and activation of the transcription factors IRF3 and NF-κB, culminating in the production of IFN-α/β and inflammatory cytokines (13). Because of its ubiquitin ligase activity, TRIM25 is considered an interferon-stimulated gene (ISG), and its gene expression is induced by type I IFNs as part of a positive feed-forward loop (14). TRIM25 regulates retroviral proliferation at different steps of the viral replication cycle (15) and inhibits the entry of the murine leukemia virus (16). The overexpression of TRIM25 in HEK293T cells reduced feline leukemia virus (FeLV) RNA and Gag protein expression by a FeLV variant (17). Zinc-finger antiviral protein (ZAP) is a host factor, the replication of many viruses can be inhibited by ZAP binding to viral mRNAs. It has been reported that TRIM25 mediated ZAP ubiquitination and synergized with it to inhibit Sindbis virus replication (18). However, the role of TRIM25 in DTMUV has not been analyzed.

In this study, we constructed the pcDNA3.1-c-myc-duTRIM25 plasmid and investigated its antiviral effect on DTMUV infection. These findings may provide new insights into pathogen-host interactions and therapeutic strategies for DTMUV infection.

## Materials and Methods

### Cell Culture and DTMUV Preparation

293T, BHK-21, and DF-1cells, and duck embryo fibroblasts (DEFs) were cultured in Dulbecco's Modified Eagle's Medium (DMEM, Gibco, Grand Island, NY, USA), which complemented with 10% fetal bovine serum (Gibco, USA) and 1% streptomycin-penicillin- (Gibco, USA) in a humidified incubator with 5% CO_2_ at 37°C. The DTMUV JS804 strain (GenBank Accession No.JF895923) was propagated in DF-1 cells to a titer of 10^5.5^ TCID_50_/0.1ml and saved in our laboratory.

### Cloning and Sequence Analysis of Duck TRIM25

The duck TRIM25 (duTRIM25) ORF (GenBank No. KY974316) was synthesized by GenScript (Nanjing, China) according to a specifically designed and codon-optimized sequence. DNA fragments were sub-cloned into the plasmid vector pcDNA3.1-c-myc (Life Technologies) utilizing the *Eco*R I/*Xho*I restriction enzymes. The amino acid sequences of bird, fish, and mammalian TRIM25s were compared using CLUSTALW. Phylogenetic trees were constructed using the neighbor-joining (NJ) method with MEGA software version 7.0. After that, to determine the confidence of tree branch positions, bootstrap analysis of 1,000 replicates was performed.

### Real-Time PCR

Gene expression levels were determined by quantitative real-time PCR using the SYBR Premix Ex Taq^TM^ II Kit (Takara, China) on the LightCycler 480 Real-Time PCR System (Roche). Specific primers were designed using Lasergene sequence analysis software (DNAStar, Inc., Madison, WI, USA) based on GenBank sequences (Table 1). Amplification was performed in 20 μl reactions containing 1 μl of cDNA and 0.8 μl of each specific primer. The following amplification conditions were used: initial denaturation at 95°C for 30 s, followed by 40 cycles at 95°C for 10 s and annealing and extension at 55°C for 45 s. Melting curves were obtained, and the relative gene expression levels were measured using the 2^−ΔΔCt^ method. Mock-infected cells were introduced as control (relative expression =1). Experiments were performed in triplicate.

**Table 1:** Primers used in quantitative real-time PCR and small interfering RNAtransfection assays.

**Gene symbol**	**Forward primer sequence (5^**′**^-3^**′**^)**	**Reverse primer sequence (5^**′**^-3^**′**^)**
qTRIM25 (duck)	CCAAGGCTGAAGCTGAGTCA	CTCAGCAGTTTGGGTTTGGC
qGAPDH (duck)	ATGTTCGTGATGGGTGTGAA	CTGTCTTCGTGTGTGGCTGT
qTRIM25 (mouse)	AGTCTGAGGAGCACAATGGC	TCCAAAGGTGGGCAACTTGT
qGAPDH (mouse)	GGCAAGTTCAAAGGCACAGTC	CACCAGCATCACCCCATTT
qTRIM25 (chicken)	CGCAGCACAACAAGCTCTTT	TGCCAGCCTCTTCTTCAGTG
qβ-actin (chicken)	ATTGTCCACCGCAAATGCTTC	AAATAAAGCCATGCCAATCTCGTC
qTRIM25 (human)	GACCACGGCTTTGTCATCTTC	AAAGTCCACCCTGAACTTATACATCA
qGAPDH (human)	GGTGGTCTCCTCTGACTTCAAGA	GTTGCTGTAGCCAAATTCGTTGT
TRIM25-gallus-si	GCGAGAUUUGCUGAGAGCUGAGUUU	AAACUCAGCUCUCAGCAAAUCUCGC
TRIM25-gallus-1412	GCUAACGUCACGCUGGAUUTT	AAUCCAGCGUGACGUUAGCTT
DTMUV-E	CGCTGAGATGGAGGATTATG	ACTGATTGTTTGGTGGCGTG

### Immunofluorescence Assay

DEFs were transferred to 24-well plates, transfected with 1 μg pcDNA3.1-c-myc-duTRIM25for 4 h using Lipofectamine 2000 (Invitrogen),and cultured in DMEM supplemented with 2% FBS (Gibco) for 24 h. Transfected DEFs were infected with DTMUV. At 24 hpi, the DTMUV replication level was analyzed by immunofluorescence and qPCR. The primers used in qPCR are listed in Table 1. For immunofluorescence, cells were fixed and permeabilized in ice-cold methanol for 10 min at −20°C, washed in PBS, and blocked with 1% bovine serum albumin. After that, cells were incubated with primary antibodies at 37°C for 2 h, washed three times with PBS, and incubated with the relevant Alexa Fluor-conjugated secondary antibodies for 1 h at room temperature. 1 μg/ml 4′, 6-diamidino-2-phenylindole (DAPI) was introduced to stain nuclei. With the application of an inverted fluorescence microscope, samples were observed and analyzed.

### Western Blotting

DEFs were lysed in radio immunoprecipitation assay buffer supplemented with protease inhibitors (Sigma-Aldrich) on ice for 30 min. Proteins were resolved by 12% SDS-PAGE and transferred to nitrocellulose membranes (Millipore). Immunoblotting was performed as described previously (19). Immunoreactive bands were visualized and analyzed using commercial enhanced ECL chemiluminescence (Beyotime, China).

### siRNA Transfection

All small interfering RNAs (siRNAs) were designed and synthesized with the help of GenePharma (Shanghai, China). Before siRNA transfection, DEFs were seeded at 1 × 10^5^ cells/well in 24-well plates for 24 h. siRNAs (25 nM) were diluted in Opti-MEM (Hyclone) in a final volume of 50 μl and mixed with 50 μl of Opti-MEM supplemented with 1.5 μl of Lipofectamine 2000 reagent (Invitrogen, Carlsbad, CA)in each well. At room temperature, the siRNA-Lipofectamine mix was incubated for 20 min firstly, and then, each well was added 400 μl Opti-MEM supplemented with 10% of FBS Subsequent experiments were performed 48 h after transfection. The siRNA sequences for duTRIM25 are listed in Table 1.

### Virus titration

Total viruses from supernatants and cell lysates were collected and titrated by plaque assay in triplicate. Monolayers of BHK-21 cells were prepared. Cells were infected with10-fold serial dilutions of the viral inoculums, and viral adsorption was performed in 1% FBS at 37°Cfor 90 min. The viral inoculum was removed, and 2% low-melting-point agarose and complete 2× DMEM (ratio of 1:1) were added. The cells were stained with crystal violet and observed under a light microscope.

### Statistical Analyses

Statistical analysis was performed using GraphPad Prism version 7.0 (GraphPad Software Inc, USA). Data were presented as means ± SEM. Comparisons between the treated and control groups were performed using Student's *t*-test. A value of *p* < 0.05 was considered statistically significant.

## Results

### Analysis of duTRIM25 Sequence

**Figure 1:**
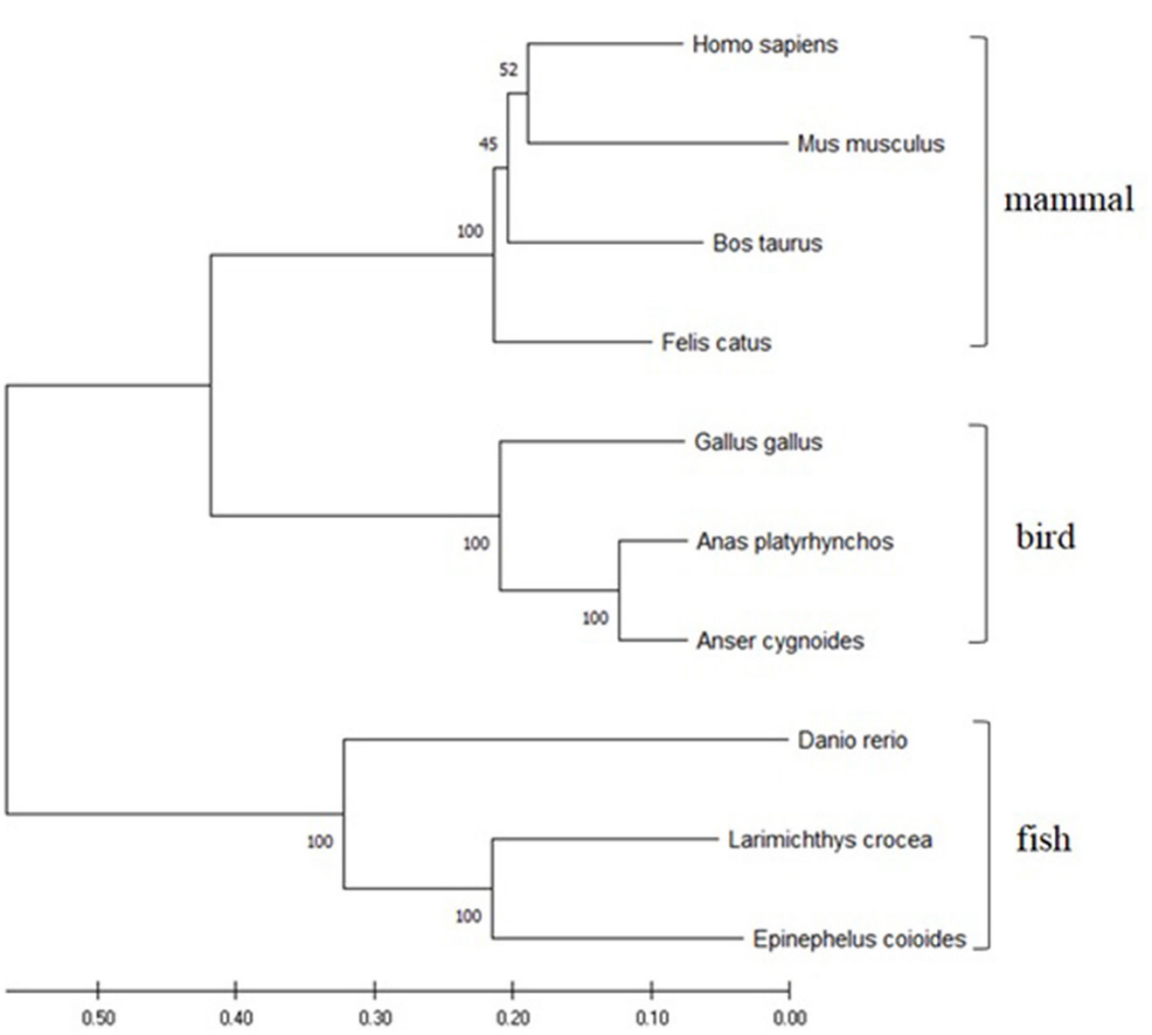
A phylogenetic tree of TRIM25 sequences was generated using MEGA 7.0 and the neighbor-joining method. The sequences were obtained from GenBank: *Homo sapiens* (NM_005082.5), *Mus musculus* (NM_009546.2), *Danio rerio* (NM_200175.1), *Gallus gallus* (NM_001318458.1), *Felis catus* (NM_001290251.1), *Bos taurus* (NM_001100336.1), *Larimichthys crocea* (MK327541.1), *Epinephelus coioides* (KX258199.1) and *Anser cygnoides* (XM_013178947).

The ORF of duTRIM25 contains 2,052 bp and encodes a predicted 683 amino acid protein. The protein sequence identity with goose, chicken, mouse, and human TRIM25 is 90.6, 69.7, 47.8, and 48.3%, respectively. Phylogenetic analysis revealed that duTRIM25 clustered into the bird clade, closer to *Anas cydnoidies* TRIM25 and *Gallus gallus* TRIM25 (Figure 1).

### Tissue Distribution Profile of duTRIM25

**Figure 2:**
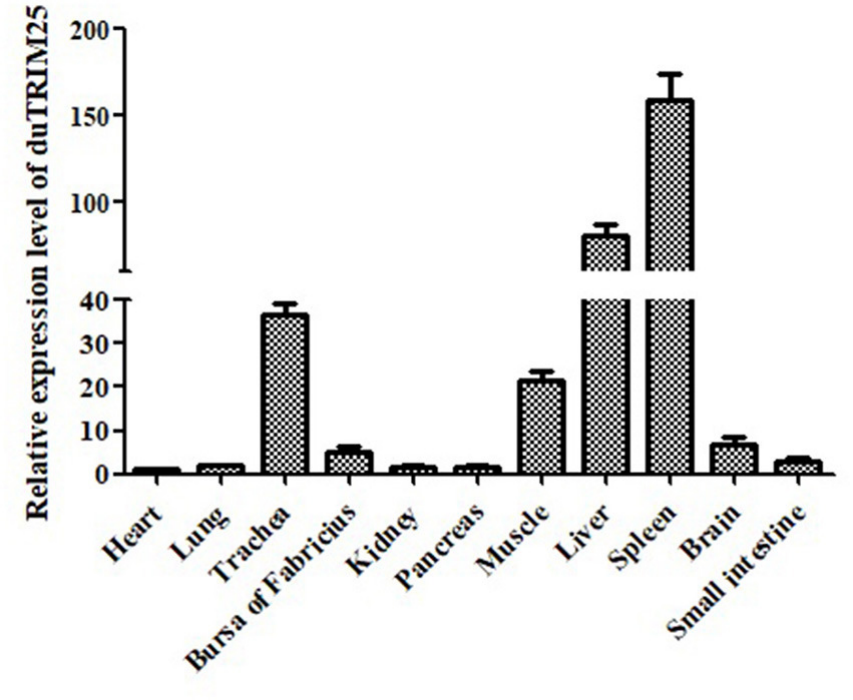
Relative expression levels of duTRIM25 in healthy ducks. The transcription levels of duTRIM25 in different tissues were measured by quantitative real-time PCR. GAPDH was used as the control gene. Data were analyzed using GraphPad Prism software, and the results are means ± SD of three independent experiments.

The expression of TRIM25 in different tissues of healthy ducks was determined by qRT-PCR, and GAPDH is used as an internal control gene. The expression of duTRIM32 was highest in the spleen, followed by the liver, trachea, muscle, brain, and Bursa of Fabricius (Figure 2), demonstrating that duTRIM25 was ubiquitously expressed in all analyzed tissues.

### TRIM25 Is Upregulated Upon DTMUV Infection

**Figure 3:**
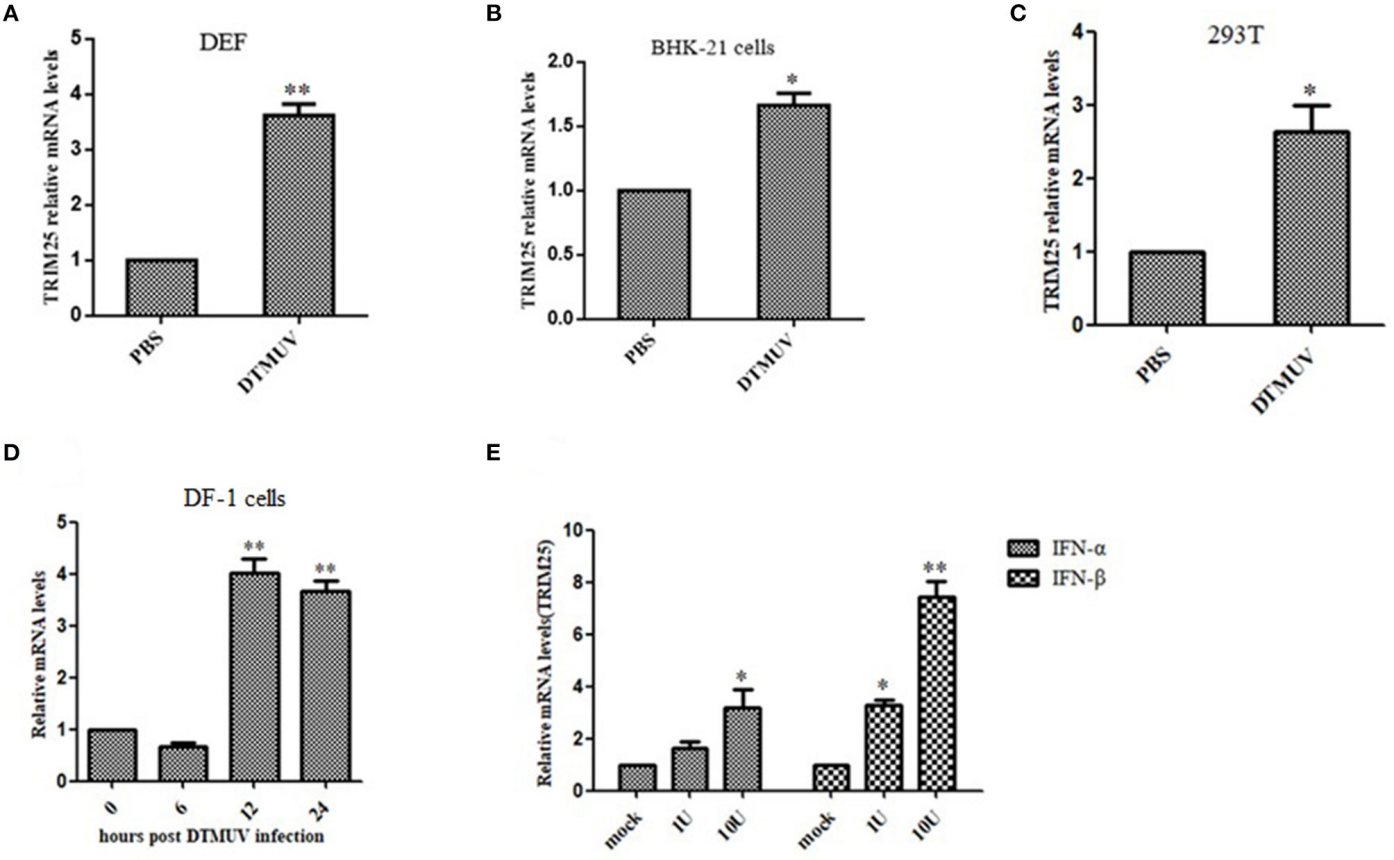
TRIM25 is induced by duck Tembusu virus (DTMUV) infection or IFN stimulation. qRT-PCR analysis of TRIM25 mRNA expression in duck embryo fibroblasts **(A)**, BHK-21 cells **(B)**, 293T cells **(C)**,and DF-1 cells **(D)** at 24 h post-infection (hpi) with DTMUV at a multiplicity of infection (MOI) of one. Cells were harvested at 0, 6, 12, and 24 hpi. **(E)** qRT-PCR analysis of TRIM25 mRNA expression in BHK-21 cells upon stimulation with IFN-α or IFN-β. The expression levels were normalized to GAPDH or β-actin and expressed as fold-change. Data are means ± SEM of three independent experiments.

TRIM25 was upregulated in DEF, BHK-21, DF-1, and 293T cells infected with DTMUV (Figures 3A–D). A previous study measured the expression of TRIM genes in response to IFNs (20). Consistent with these conclusions, we found that the mRNA expression of TRIM25 was induced in BHK-21 cells upon stimulation with IFN-α and IFN-β (Figure 3E). These results confirm that TRIM25 is an ISG induced by type I IFN and viral infection.

### The Overexpression of duTRIM25 Inhibits DTMUV Replication

**Figure 4:**
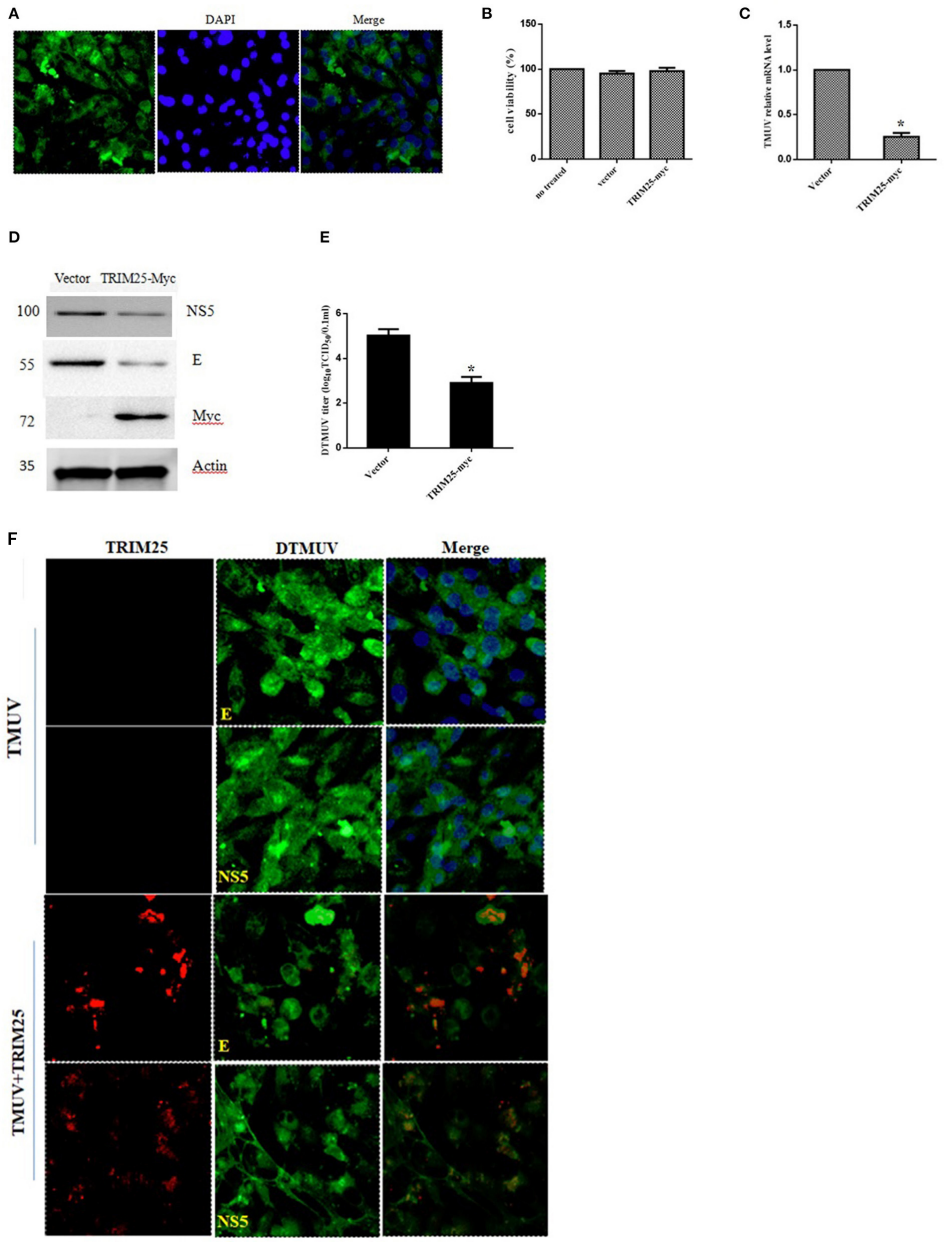
The replication of duck Tembusu virus (DTMUV) is limited by TRIM25. **(A)** Expression of TRIM25 by immunofluorescence. Duck embryo fibroblasts (DEFs) were transiently transfected with the pcDNA3.1-c-myc-TRIM25 plasmid. Nuclei were stained with DAPI. **(B)** Cell viability assays after transfection with TRIM25 or empty vector. **(C–F)** TRIM25 ectopic expression inhibited DTMUV replication. The expression of viral RNA **(C)** and proteins **(D)** was analyzed in DTMUV-infected DEFs with or without TRIM25 overexpression. **(E)** Titers of DTMUV in the supernatants of infected DEFs with or without TRIM25 transfection were measuredusingTCID_50_ assays. **(F)** Immunofluorescence analysis of DTMUV proteins in DEFs. DEFs were transfected with TRIM25 or an empty vector for 24 hand infected with DTMUV for 24 h. Immunofluorescence was performed using E/NS5 mouse polyclonal antibody and c-myc mouse antibody.

TRIM25 plays a crucial role in the RIG-I-mediated activation of type I IFN and MAVS ubiquitination and degradation (15). Herein, we constructed the recombinant plasmid pcDNA3.1-c-myc-duTRIM25 and transfected DEFs with this plasmid. Immunofluorescence showed that the plasmid was stably expressed in DEFs (Figure 4A). DEFs were transfected with pcDNA3.1-c-myc-duTRIM25 and infected with DTMUV to explore the function of duTRIM25 in DTMUV replication. The transient overexpression of TRIM25 did not cause distinct cell toxicity at 60 hpi (Figure 4B). Both qRT-PCR and western blot results suggested that viral RNA and proteins significantly decreased in TRIM25-overexpressed cells compared with mock-infected cells (Figures 4C,D). Consistent with these results, viral load in cell supernatants was lower in TRIM25-overexpressed cells (Figure 4E), and immunofluorescence confirmed that viral E and NS5 protein levels significantly decreased in these cells (Figure 4F).

### The siRNA-Mediated Knockdown of TRIM25 Increases DTMUV Infection

**Figure 5:**
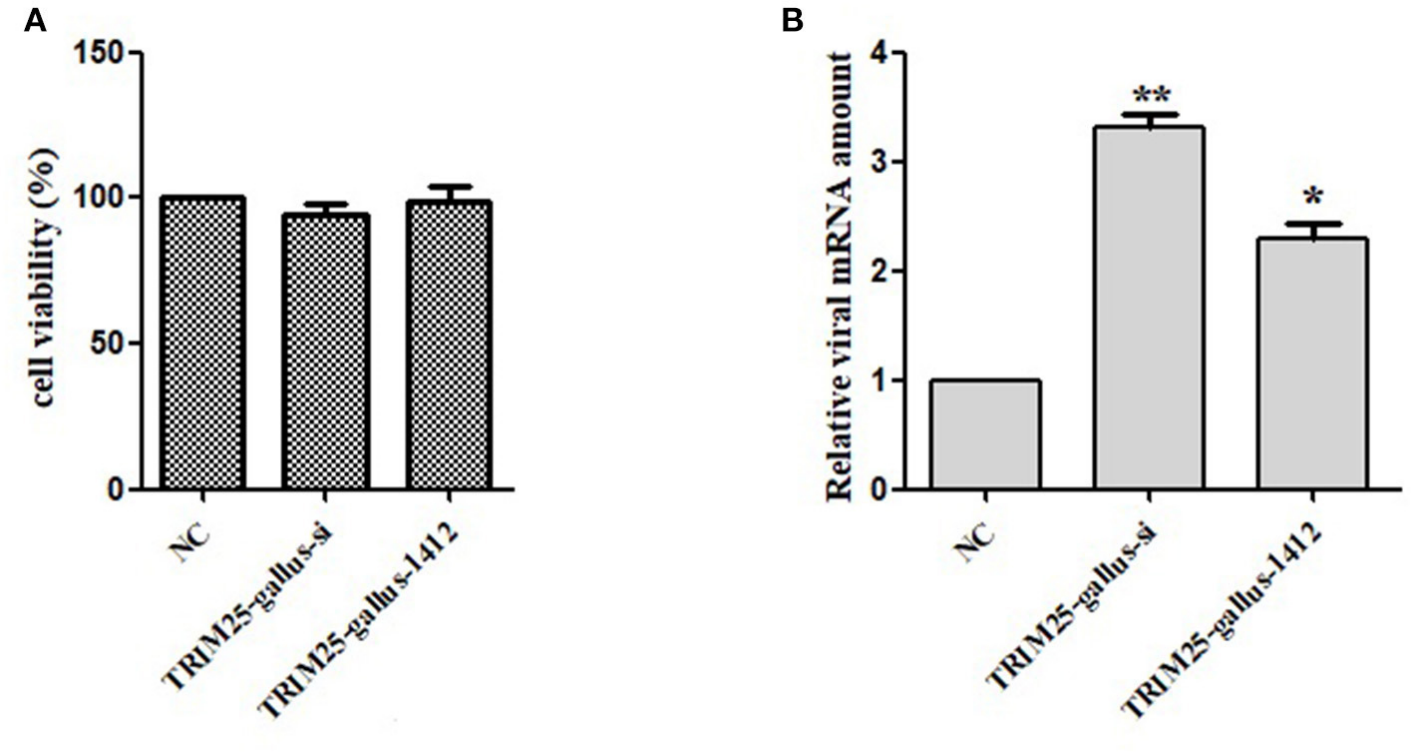
Knockdown of TRIM25 by siRNA increases duck Tembusu virus (DTMUV) infection. **(A)**TRIM25 knockdown did not cause cell toxicity. **(B)** DTMUV mRNA expressioninTRIM25-silenced (TRIM25-gallus-si and TRIM25-gallus-1412) duck embryo fibroblasts and control cells the relative amount of DTMUV mRNA was analyzed.

Two TRIM25siRNAs (TRIM25-gallus-si and TRIM25-gallus-1412) were designed to confirm this phenotype (Table 1). Silencing TRIM25did not influence cell viability (Figure 5A). The relative mRNA expression of DTMUV significantly increased in two TRIM25 siRNA-transfected DEF lineages after DTMUV infection (Figure 5B).

## Discussion

DTMUV is a viral pathogen that infects waterfowl, including ducks and geese, and has posed a significant threat to the domestic poultry industry since 2010 (1, 3, 21). In a previous study, we performed transcriptional analysis to obtain global information on DF-1 cells infected with DTMUV and found that 663 genes were differentially expressed (73 down regulated and 590 up regulated) (10). TRIM25 was differentially expressed and chosen for further analysis on DTMUV.

TRIM25 belongs to the TRIM protein family and is characterized by a conserved N-terminus domain (known as the TRIM or RBCC motif) composed of a catalytic RING domain, one or two B-box domains, also contain a coiled-coil dimerization domain, as well as a C-terminal PRY/SPRY domain (22). The RING domain functions as an E3 ligase, and the C-terminal domain targets proteins for TRIM ubiquitination (23). The lysine residues 63-linked ubiquitination of the N-terminal CARD domains of the viral RNA sensor RIG-I was mediated by TRIM25 firstly, and then facilitated type I IFN production and antiviral immunity (24). TRIM25 is ubiquitous in nature because of its crucial role and has been found in fish, humans, mice, and birds. We found that duTRIM25 was ubiquitously expressed in the spleen, liver, trachea, muscle, brain, and Bursa of Fabricius of healthy ducks. Conversely, TRIM25 transcription was highest in the blood of gosling and adult goose (25) and in the lungs of *G. gallus* (26). Phylogenetic analysis revealed that duTRIM25clustered into the bird clade and was homologous to TRIM25 from goose (up to 90.6%), mouse (47.8%), and human (48.3%).

The results showed that duTRIM25 was upregulated in BHK-21 and 293T cells and DEFsby DTMUV infection, and previous RNA-seq analysis indicated that duTRIM25 was also induced by type I IFN. Such as TRIM69, TRIM14, and TRIM52 exist antiviral activity have been proved (20, 27, 28). We constructed the pcDNA3.1-c-myc-duTRIM25 plasmid to investigate the mechanism by which TRIM25 inhibits DTMUV replication. The plasmid was transfected into DEFs, and mRNA expression of DTMUV was measured by qRT-PCR. The results showed that the overexpression of TRIM25 inhibited the replication of DTMUV (Figure 4C). Western blot and immunofluorescence results confirmed that viral E and NS5 protein levels decreased in TRIM25-overexpressed DEFs significantly (Figures 4D,F). When silencing TRIM25 by siRNA transfection, the relative amount of DTMUV mRNA increased significantly in two TRIM25 siRNAs transfected DEFs after DTMUV infection (Figure 5). TRIM25 covalently attaches K63-linked polyubiquitin to CARDs of RIG-I in order to initiate or promote signaling. Moreover, it has been reported that the melanoma differentiation-associated protein 5 (MDA5)-MAVS-TRAF6 antiviral axis could be also positively regulated by TRIM25, leading to the activation of NF-κB (29). Besides involved in its ubiquitin ligase activity and the interferon pathway, TRIM25 plays antiviral effects via other pathways. ZAP is a host factor that inhibits viral replication by binding to viral mRNAs and repressing the translation and/or promoting the degradation of target mRNAs. Li et al. reported that ZAP ubiquitination could be mediated by TRIM25 and synergized with TRIM25 to inhibit Sindbis virus replication (18). TRIM25 targets influenza ribonucleoproteins (RNPs) directly, thereby inhibits viral mRNA synthesis, which is catalyzed by the tripartite viral polymerase associated with these proteins (30). In addition, the ubiquitin-like protein ISG15 has been reported to conjugate TRIM25 to target proteins together in a process known as ISGylation (31). ISGylation and free ISG15 have broad-spectrum antiviral effects (32–34). These data indicate that TRIM25 acts on multiple signaling pathways. Our results showed that TRIM25 inhibited DTMUV replication in host cells. Nonetheless, the mechanism by which TRIM25 targets DTMUV needs to be further investigated.

In summary, we analyzed the duck TRIM25 gene and constructed the pcDNA3.1-c-myc-duTRIM25 plasmid to investigate the antiviral effect of TRIM25 on DTMUV infection. We found that duTRIM25 inhibited DTMUV infection *in vitro*. These results help elucidate the function of duTRIM proteins in regulating the type I IFN pathway and their antiviral approach in DTMUV control.

## Data Availability Statement

The original contributions presented in the study are included in the article/supplementary material, further inquiries can be directed to the corresponding author/s.

## Author Contributions

HK, DZ, YZL, and QL designed research. HK, DZ, YZL, QL, XH, JY, LZ, and YL performed research. JY, LZ, and YL analyzed data. HK and DZ drafted the manuscript. All authors read and approved the final manuscript.

## Funding

This work was supported by Jiangsu Provincial Agricultural Science and Technology Innovation Foundation [No. cx (20) 3093], National Natural Science Foundation of China (No. 31502101), and National Key Research and Development Program of China (2017YFD0500804).

## Conflict of Interest

The reviewer LM declared a shared affiliation, though no collaboration, with the authors, HK, ZD, LY, LQ, HX, YJ, ZL, and LY at the time of review.

## Publisher's Note

All claims expressed in this article are solely those of the authors and do not necessarily represent those of their affiliated organizations, or those of the publisher, the editors and the reviewers. Any product that may be evaluated in this article, or claim that may be made by its manufacturer, is not guaranteed or endorsed by the publisher.
